# Supplementary material for: An Explanation about the Use of (S)-Citronellal as a Chiral Derivatizing Agent (CDA) in 1H and 13C NMR for Sec-Butylamine, Methylbenzylamine, and Amphetamine: A Theoretical-Experimental Study
Source: Molecules. 2019 Aug 3;24(15):2830. doi: 10.3390/molecules24152830 (PMC6695982; doi:10.3390/molecules24152830)
Supplement: Supplementary file 1 [file molecules-24-02830-s001.pdf]

Supplementary Information

An explanation about the use of (*S*)-citronelal as chiral derivatizing agent (CDA) in ^{1}H and ^{13}C NMR for *sec*-butylamine, methylbenzylamine, and amphetamine: a theoretical-experimental study

Viviani Nardini*, Vinicius Palaretti, Luis Gustavo Dias and Gil Valdo José da Silva

Departamento de Química, Faculdade de Filosofia, Ciências e Letras de Ribeirão Preto, Universidade de São Paulo, Avenida dos Bandeirantes, 3900, 14040-901 Ribeirão Preto, SP, Brazil, Tel.: +55-16-3315-4382; fax: +55-16-3633-8151

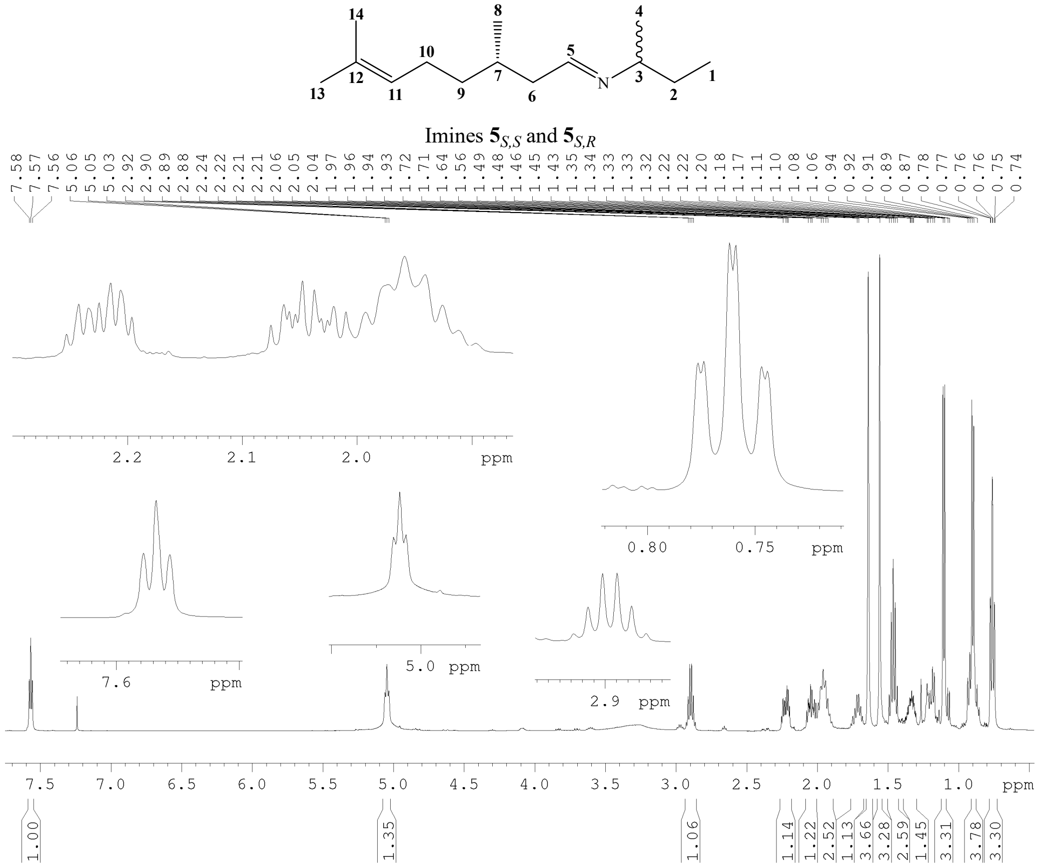

Figure S1. ^{1}H NMR spectrum of the S,S and S,R diastereoisomers derived from the reaction between (*S*)-citronellal and the racemic mixture of *sec*-butylamine (imines **5*S,S*** and **5*S,R***, $CDCl_3$, 500 MHz).

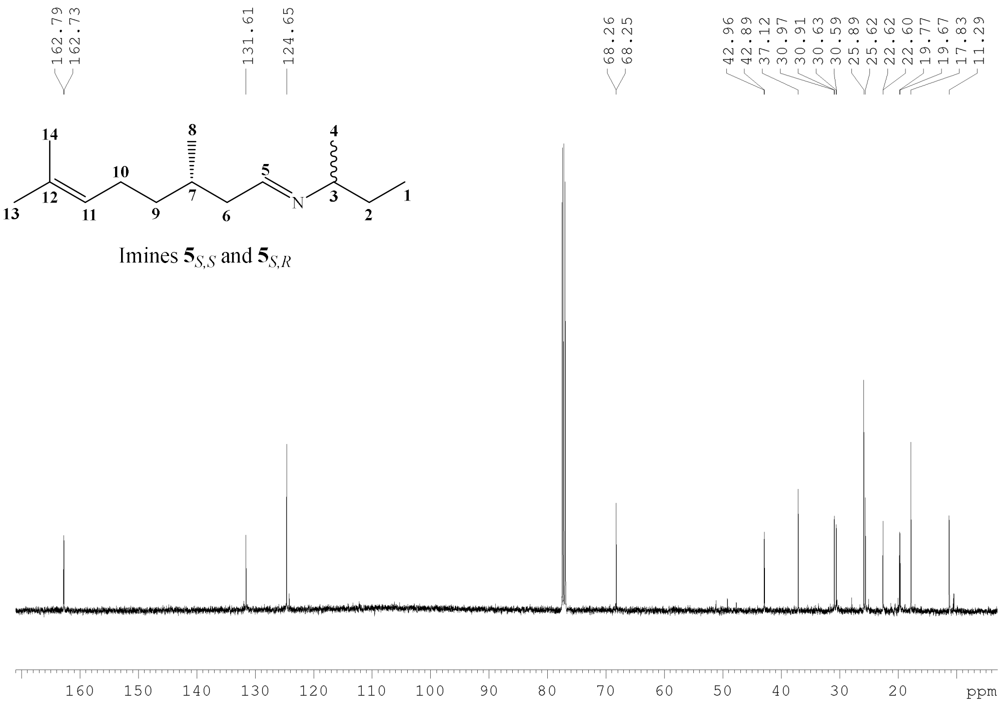

Figure S2. ^{13}C NMR spectrum of the S,S and S,R diastereoisomers derived from the reaction between (*S*)-citronellal and the racemic mixture of *sec*-butylamine (imines **5*S,S*** and **5*S,R***, $CDCl_3$, 500 MHz).

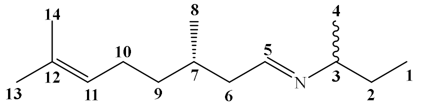

Imines $\mathbf{5}_{S,S}$ and $\mathbf{5}_{S,R}$

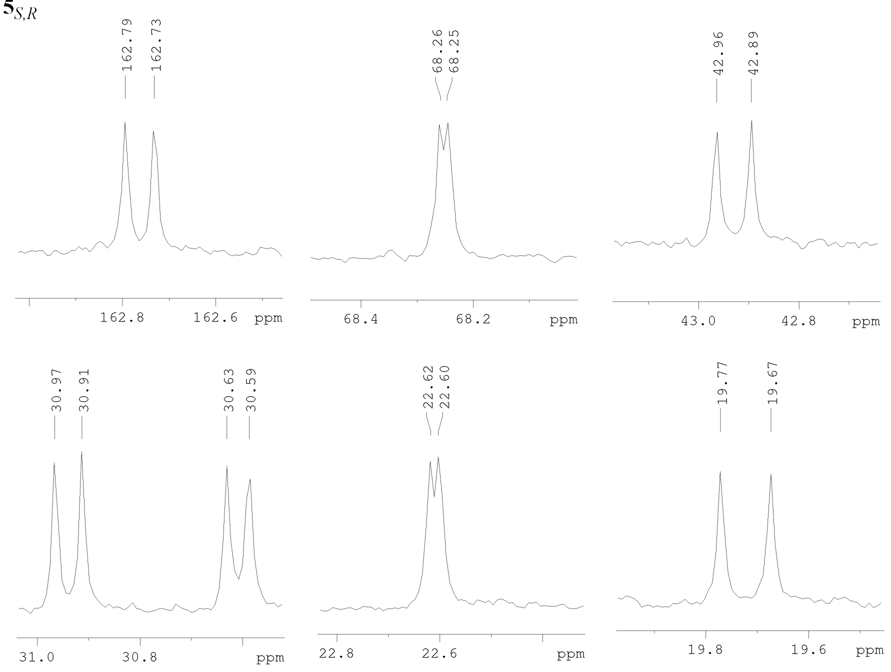

Figure S3. Expansion of ^{13}C NMR spectrum of the *S,S* and *S,R* diastereoisomers derived from the reaction between (*S*)-citronellal and the racemic mixture of *sec*-butylamine (imines $\mathbf{5}_{S,S}$ and $\mathbf{5}_{S,R}$, $CDCl_3$, 500 MHz).

Table S1. Spectroscopic data of the S,S and S,R diastereoisomers derived from the reaction between (*S*)-citronellal and the racemic mixture of *sec*-butylamine (imines $\mathbf{5}_{S,S}$ and $\mathbf{5}_{S,R}$, $CDCl_3$, 500 MHz).

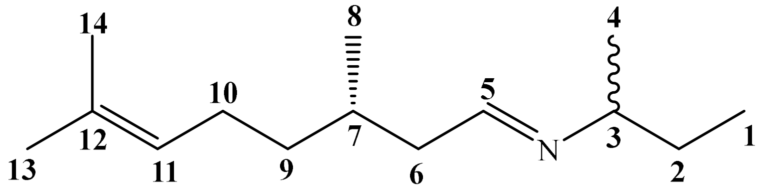

Imines $\mathbf{5}_{S,S}$ and $\mathbf{5}_{S,R}$

Number	H (ppm)	Multiplicity	*J* (Hz)	*δ*C (ppm)
1	0.76	2 triplets	7.4	(*S,R*) 11.29 and 11.25 (*S,S*)
2	1.46	Overlapping signals		(*S,R*) 30.63 and 30.59 (*S,S*)
3	2.90	Overlapping signals		(*S,R*) 68.26 and 68.25 (*S,S*)
4	1.10	Overlapping signals		(*S,S*) 22.62 and 22.60 (*S,R*)
5	7.57	Overlapping signals		(*S,R*) 162.79 and 162.73 (*S,S*)
6a	2.23	Overlapping signals		(*S,R*) 42.96 e 42.89 (*S,S*)
6b	2.05	Overlapping signals		
7	1.72	Overlapping signals		(*S,S*) 30.97 e 30.91 (*S,R*)
8	0.90	Overlapping signals		(*S,R*) 19.77 e 19.67 (*S,S*)
9a	1.33	Overlapping signals		37.12
9b	1.19	Overlapping signals		
10a<br>10b	1.96	Overlapping signals		25.62
11	5.05	Overlapping signals		124.65
12	-	-	-	131.61
13	1.56	Overlapping signals		25.89
14	1.64	Overlapping signals		17.83

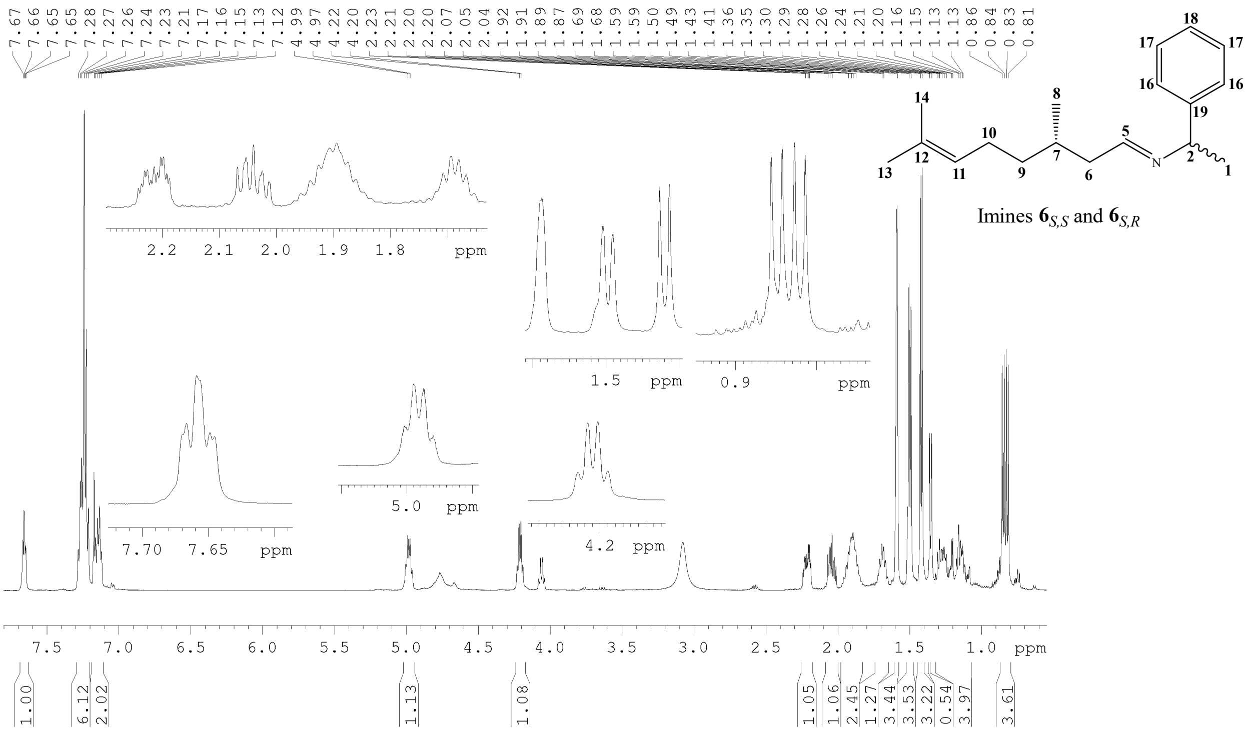

Figure S4. ^{1}H NMR spectrum of the <u>*S,S* and *S,R* diastereoisomers</u> derived from the reaction between (*S*)-citronellal and the racemic mixture of methylbenzylamine (imines $\mathbf{6}_{S,S}$ and $\mathbf{6}_{S,R}$, $CDCl_3$, 500 MHz).

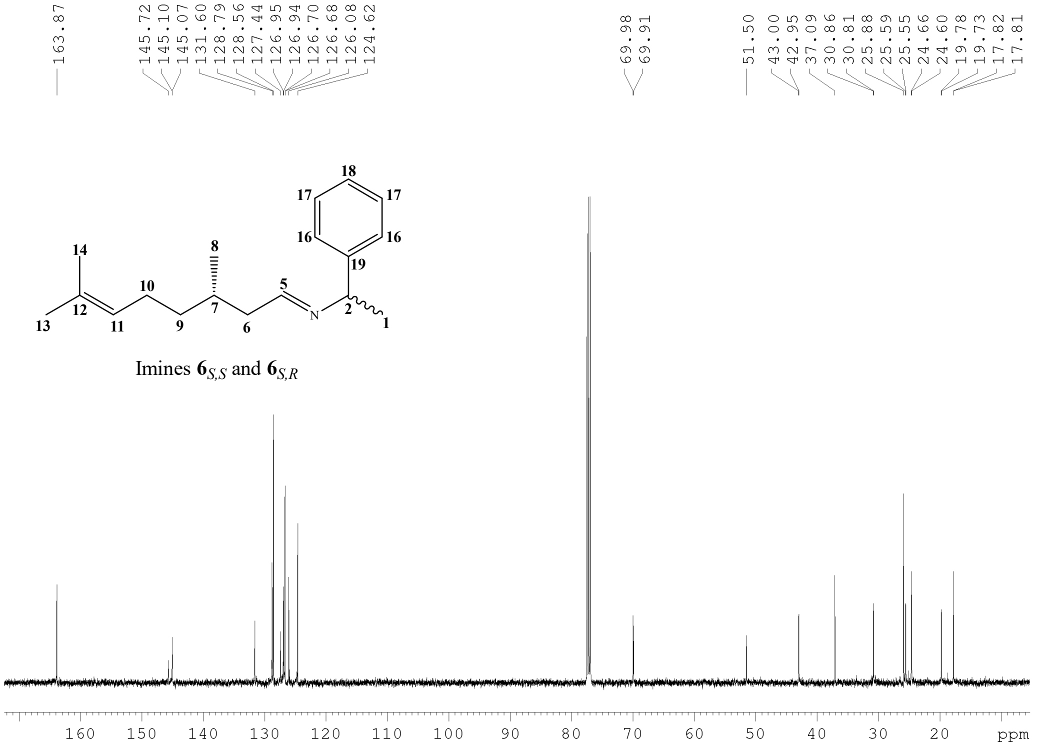

Figura S5. ^{13}C NMR spectrum of the <u>*S,S* and *S,R* diastereoisomers</u> derived from the reaction between (*S*)-citronellal and the racemic mixture of methylbenzylamine (imines **6*S,S*** and **6*S,R***, $CDCl_3$, 500 MHz).

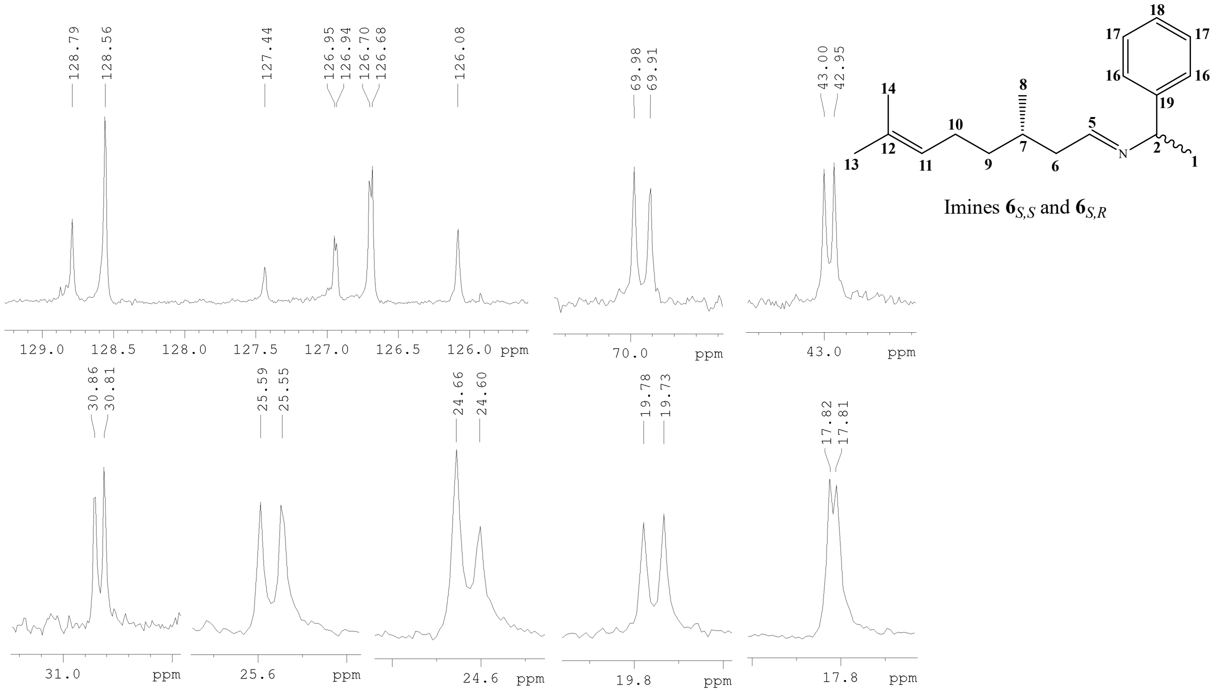

Figura S6. Expansions of ^{13}C NMR spectrum of the <u>*S,S* and *S,R* diastereoisomers</u> derived from the reaction between (*S*)-citronellal and the racemic mixture of methylbenzylamine (imines **6*S,S*** and **6*S,R***, $CDCl_3$, 500 MHz).

Table S2. Spectroscopic data of the *S,S* and *S,R* diastereoisomers derived from the reaction between (*S*)-citronellal and the racemic mixture of methylbenzylamine (imines $\mathbf{6}_{S,S}$ and $\mathbf{6}_{S,R}$, $CDCl_3$, 500 MHz).

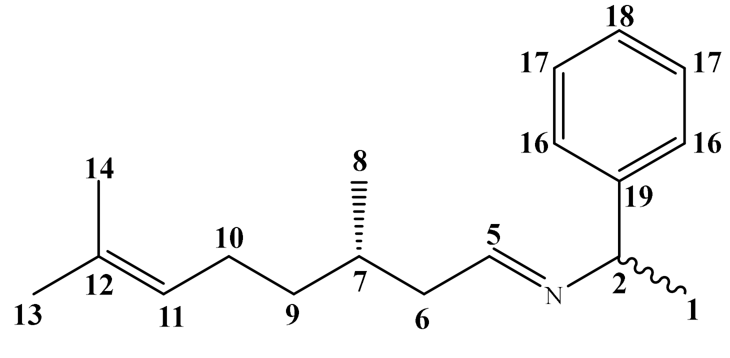

Imines $\mathbf{6}_{S,S}$ and $\mathbf{6}_{S,R}$

Number	δ_H (ppm)	Multiplicity	*J* (Hz)	δ_C (ppm)
1	1.42	2 dublets	6.3	(*S,R*) 24.66 and 24.60 (*S,S*)
2	4.21	Overlapping signals		(*S,R*) 69.98 and 69.91 (*S,S*)
5	7.66	Overlapping signals		163,87
6a	2.21	Overlapping signals		(*S,S*) 43.00 and 42.95 (*S,R*)
6b	2.04	Overlapping signals		
7	1.69	Overlapping signals		(*S,S*) 30.86 and 30.81 (*S,R*)
8	0.84	2 dublets	6.7	(*S,S*) 19.78 and 19.73 (*S,R*)
9a	1.23	Overlapping signals		37.09
9b	1.13	Overlapping signals		
10a<br>10b	1.90	Overlapping signals		(*S,S*) 25.59 and 25.55 (*S,R*)
11	4.98	Overlapping signals		124.62
12	-	-	-	131.60
13	1.50	2 singlets	-	25.88
14	1.59	2 singlets	-	17.82 and 17.81
16	Overlapping signals 7.10 to 7.30 ppm			126.95 and 126.94
17				128.56
18				126.70 and 126.68
19	-	-	-	(*S,S*) 145.10 and 145.07 (*S,R*)

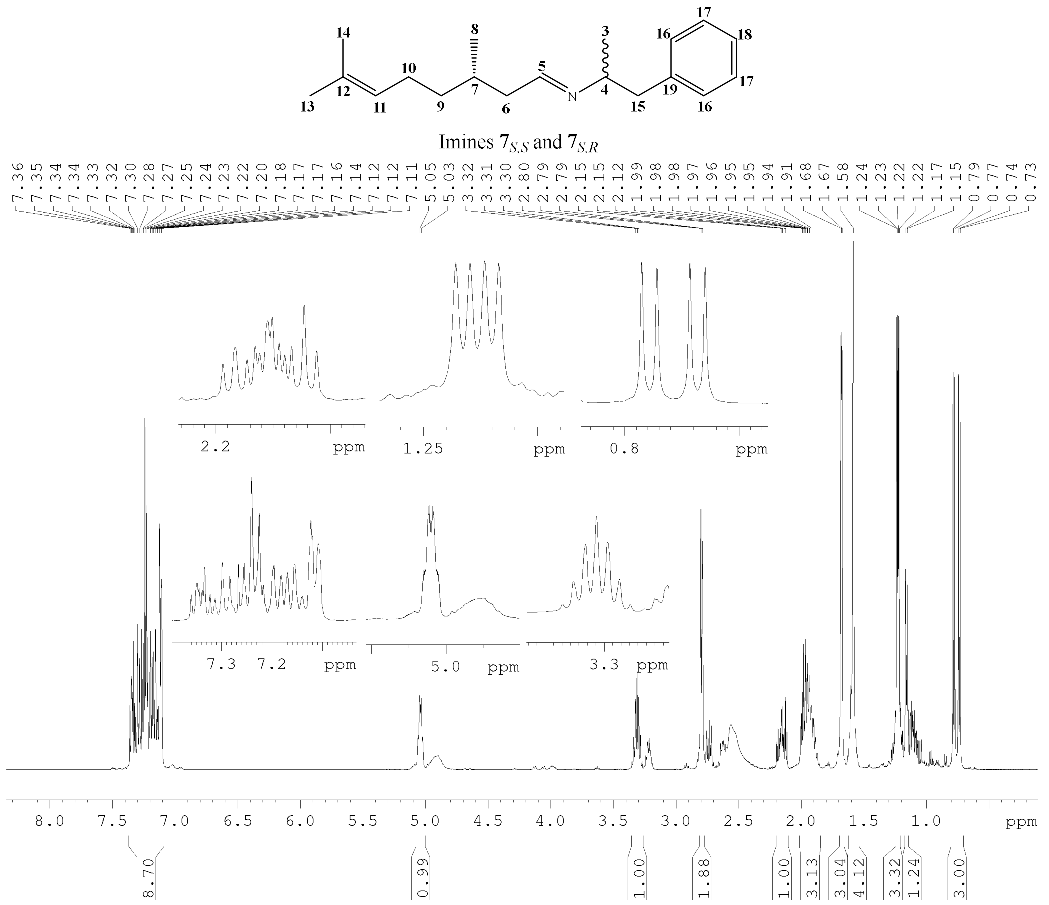

Figure S7. ^{1}H NMR spectrum of the <u>S,S and S,R diastereoisomers</u> derived from the reaction between (*S*)-citronellal and the racemic mixture of amphetamine (imines **7*S,S*** and **7*S,R***, $CDCl_3$, 500 MHz).

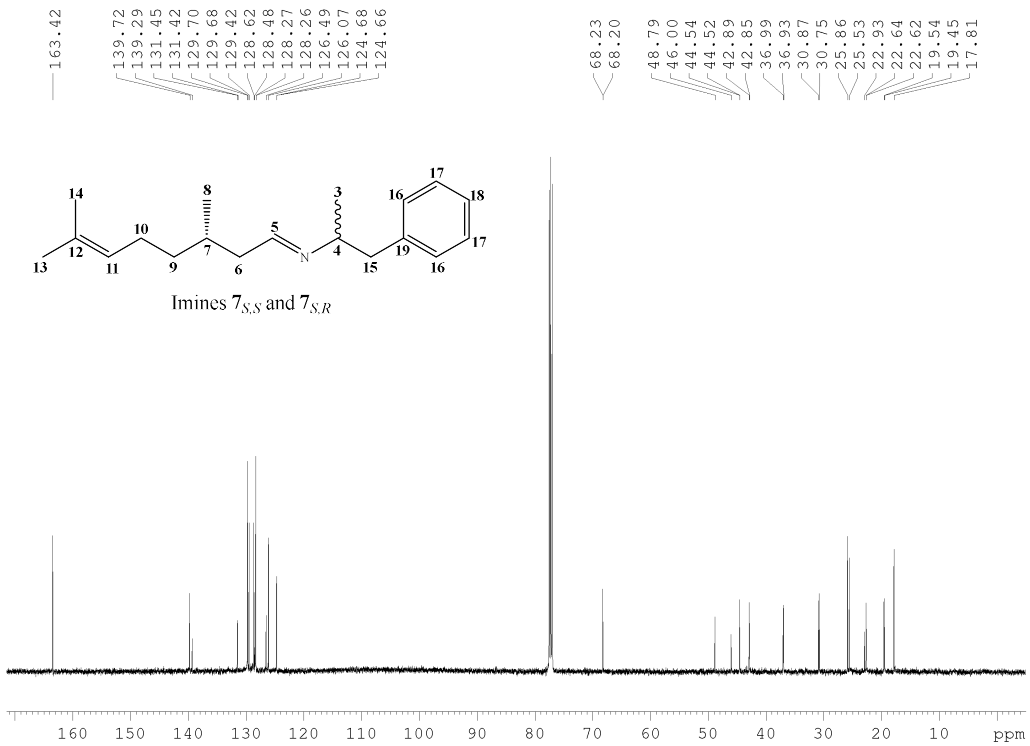

Figure S8. ^{13}C NMR spectrum of the _S,S and S,R diastereoisomers_ derived from the reaction between (*S*)-citronellal and the racemic mixture of amphetamine (imines **7*S,S*** and **7*S,R***, $CDCl_3$, 500 MHz).

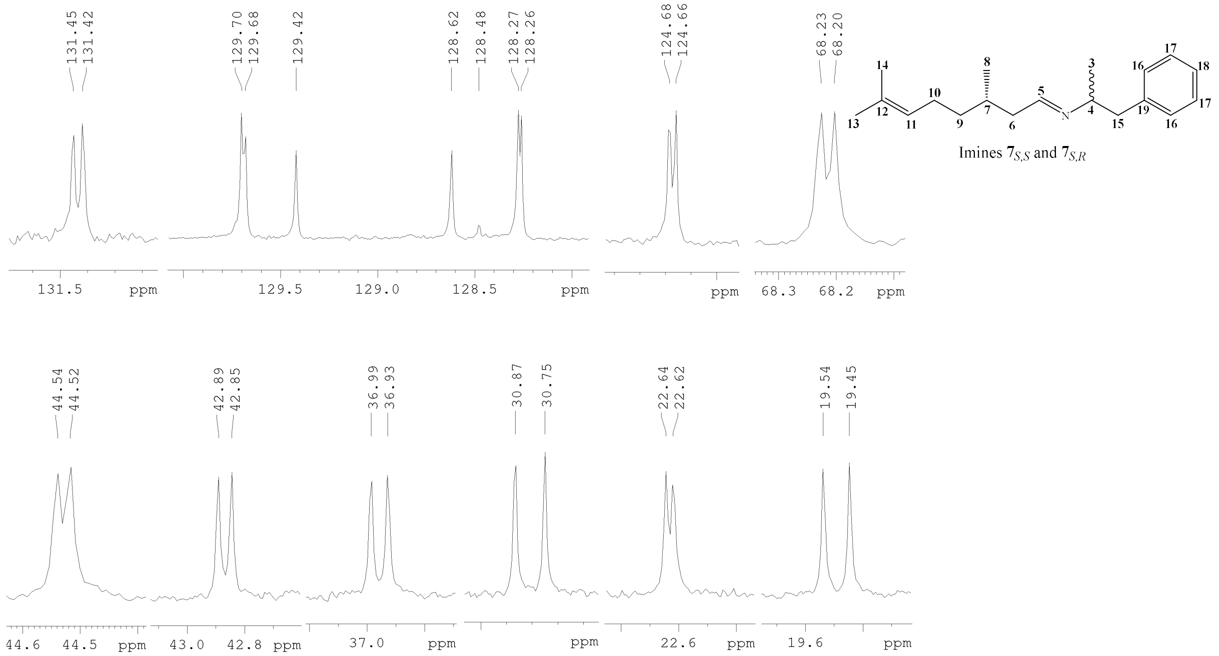

Figure S9. Expansions of ^{13}C NMR spectrum of the _S,S and S,R diastereoisomers_ derived from the reaction between (*S*)-citronellal and the racemic mixture of amphetamine (imines **7*S,S*** and **7*S,R***, $CDCl_3$, 500 MHz).

Table S3. Spectroscopic data of the *S,S* and *S,R* diastereoisomers derived from the reaction between (*S*)-citronellal and the racemic mixture of amphetamine (imines $\mathbf{7}_{S,S}$ and $\mathbf{7}_{S,R}$, $CDCl_3$, 500 MHz).

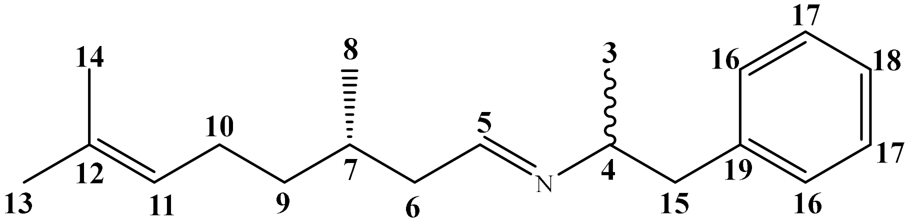

Imines $7_{S,S}$ and $7_{S,R}$

Number	δ_H (ppm)	Multiplicity	J (Hz)	δ_C (ppm)
3	1.22	2 doublets	6.3	22.64 and 22.62
4	3.28	Overlapping signals		68.23 and 68.20
5	Overlapping with H16, H17 and H18			163.42
6a	2.15	Overlapping signals		42.89 and 42.85
6b	1.98	Overlapping signals		42.89 and 42.85
7	1.60	Overlapping signals		30.87 and 30.75
8	0.75	2 doublets	6.6	19.54 and 19.45
9a	1.11	Overlapping signals		36.99 and 36.93
9b	1.25	Overlapping signals		36.99 and 36.93
10a	1.93	Overlapping signals		25.53
10b	1.93	Overlapping signals		25.53
11	5.04	Overlapping signals		124.68 and 124.66
12	-	-	-	131.45 and 131.42
13	1.58	Overlapping signals		25.86
14	1.67	2 singlets	-	17.81
15a	2.79	Overlapping signals		44.54 and 44.52
15b	2.79	Overlapping signals		44.54 and 44.52
16	Overlapping with H5 7.1 to 7.4 ppm			128.27 and 128.26
17	Overlapping with H5 7.1 to 7.4 ppm			129.70 and 129.68
18	Overlapping with H5 7.1 to 7.4 ppm			126.07
19	-	-	-	139.72

Table S4. ΔEnergy (kcal/mol) and most stable conformers distribution of the diastereoisomer **5** (*S,S* and *S,R*) using B3LYP-D3(BJ)/def2-TZVP(-f) level

Conformer	ΔEnergy (kcal/mol)	Conformer	ΔEnergy (kcal/mol)
$5_{S,S}$.1	0.00	$5_{S,R}$.1	0.00
$5_{S,S}$.2	0.20	$5_{S,R}$.2	0.14
$5_{S,S}$.3	0.27	$5_{S,R}$.3	0.16
$5_{S,S}$.4	0.38	$5_{S,R}$.4	0.87
$5_{S,S}$.5	0.43	$5_{S,R}$.5	0.88
$5_{S,S}$.6	0.63	$5_{S,R}$.6	0.92
$5_{S,S}$.7	0.88	$5_{S,R}$.7	0.97
$5_{S,S}$.8	0.89	$5_{S,R}$.8	1.11
$5_{S,S}$.9	1.06		
$5_{S,S}$.10	1.13		
$5_{S,S}$.11	1.15		
$5_{S,S}$.12	1.16		
$5_{S,S}$.13	1.20		
$5_{S,S}$.14	1.25		
$5_{S,S}$.15	1.27		
$5_{S,S}$.16	1.38		
$5_{S,S}$.17	1.39		
$5_{S,S}$.18	1.47		
$5_{S,S}$.19	1.50		
$5_{S,S}$.20	1.61		
$5_{S,S}$.21	1.61		
$5_{S,S}$.22	1.64		
$5_{S,S}$.23	1.64		
$5_{S,S}$.24	1.65		
$5_{S,S}$.25	1.70		
$5_{S,S}$.26	1.71		
$5_{S,S}$.27	1.77		
$5_{S,S}$.28	1.81		

Table S5. ΔEnergy (kcal/mol) and most stable conformers distribution of the diastereoisomer **6** (*S,S* and *S,R*) using B3LYP-D3(BJ)/def2-TZVP(-f) level

Conformer	ΔEnergy kcal/mol)	Conformer	ΔEnergy (kcal/mol)
$6_{S,R}$.1	0.00	$6_{S,S}$.1	0.00
$6_{S,R}$.2	0.06	$6_{S,S}$.2	0.68
$6_{S,R}$.3	0.12	$6_{S,S}$.3	0.87
$6_{S,R}$.4	0.26	$6_{S,S}$.4	1.08
$6_{S,R}$.5	0.26	$6_{S,S}$.5	1.10
$6_{S,R}$.6	0.59	$6_{S,S}$.6	1.33
$6_{S,R}$.7	0.84	$6_{S,S}$.7	1.43
$6_{S,R}$.8	0.93		
$6_{S,R}$.9	0.95		
$6_{S,R}$.10	0.99		
$6_{S,R}$.11	1.12		
$6_{S,R}$.12	1.15		
$6_{S,R}$.13	1.15		
$6_{S,R}$.14	1.26		
$6_{S,R}$.15	1.29		
$6_{S,R}$.16	1.34		
$6_{S,R}$.17	1.40		
$6_{S,R}$.18	1.41		
$6_{S,R}$.19	1.41		
$6_{S,R}$.20	1.50		

Table S6. ΔEnergy (kcal/mol) and most stable conformers distribution of the diastereoisomer **7** (*S,S* and *S,R*) using B3LYP-D3(BJ)/def2-TZVP(-f) level

Conformer	ΔEnergy (kcal/mol)	Conformer	ΔEnergy (kcal/mol)
$7_{S,S}$.1	0.00	$7_{S,R}$.1	0.00
$7_{S,S}$.2	0.00	$7_{S,R}$.2	0.77
$7_{S,S}$.3	0.59		
$7_{S,S}$.4	0.80		
$7_{S,S}$.5	0.91		
$7_{S,S}$.6	0.92		
$7_{S,S}$.7	1.02		
$7_{S,S}$.8	1.10		

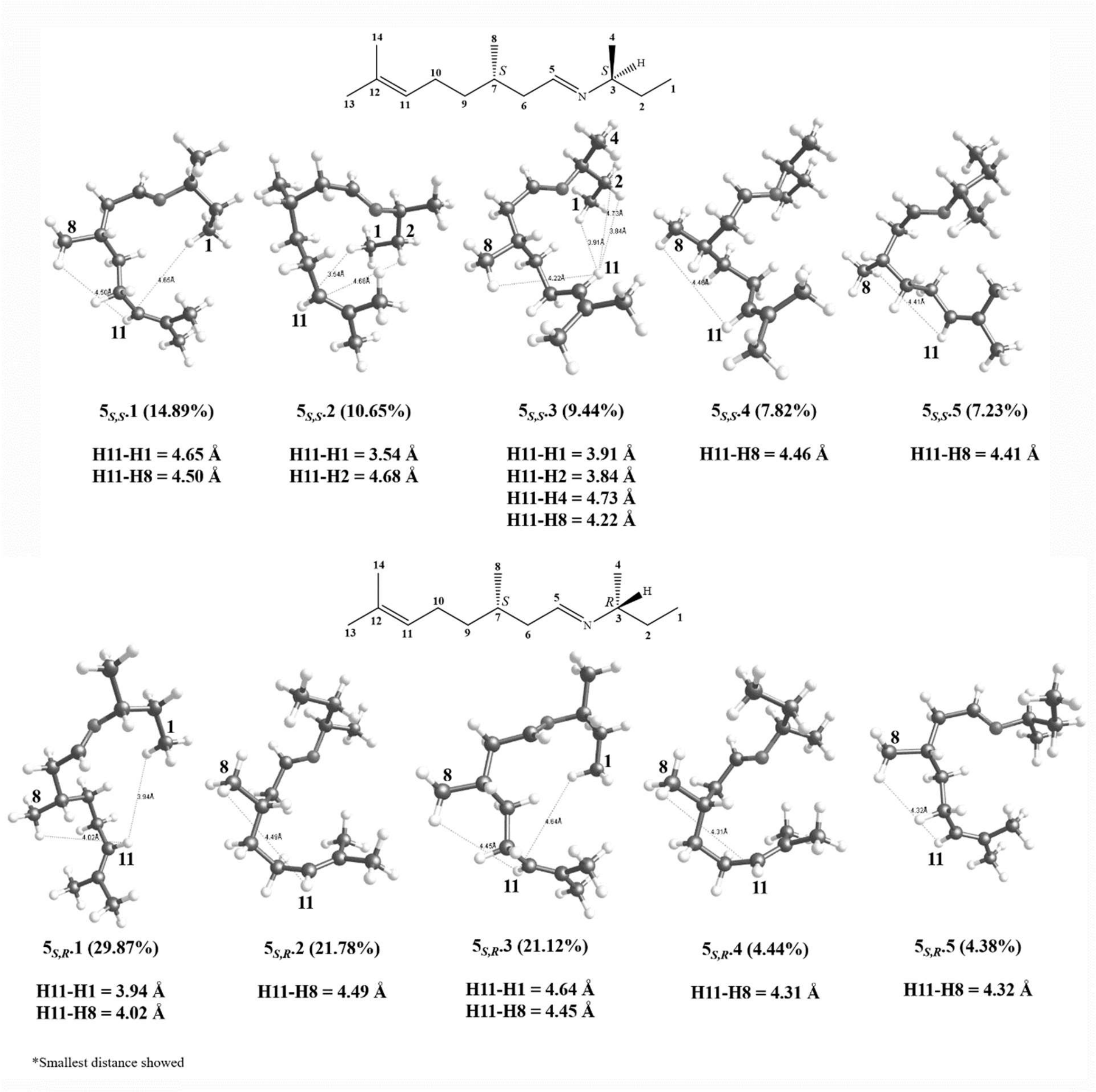

Figure S10. Five most stable conformers distribution of the diastereoisomer **5** (*S,S* and *S,R*) using B3LYP-D3(BJ)/def2-TZVP(-f) level. In parentheses is the percentage of Boltzmann's population. Just below each conformer, there is the distance, less than 5 Å, between the hydrogens.

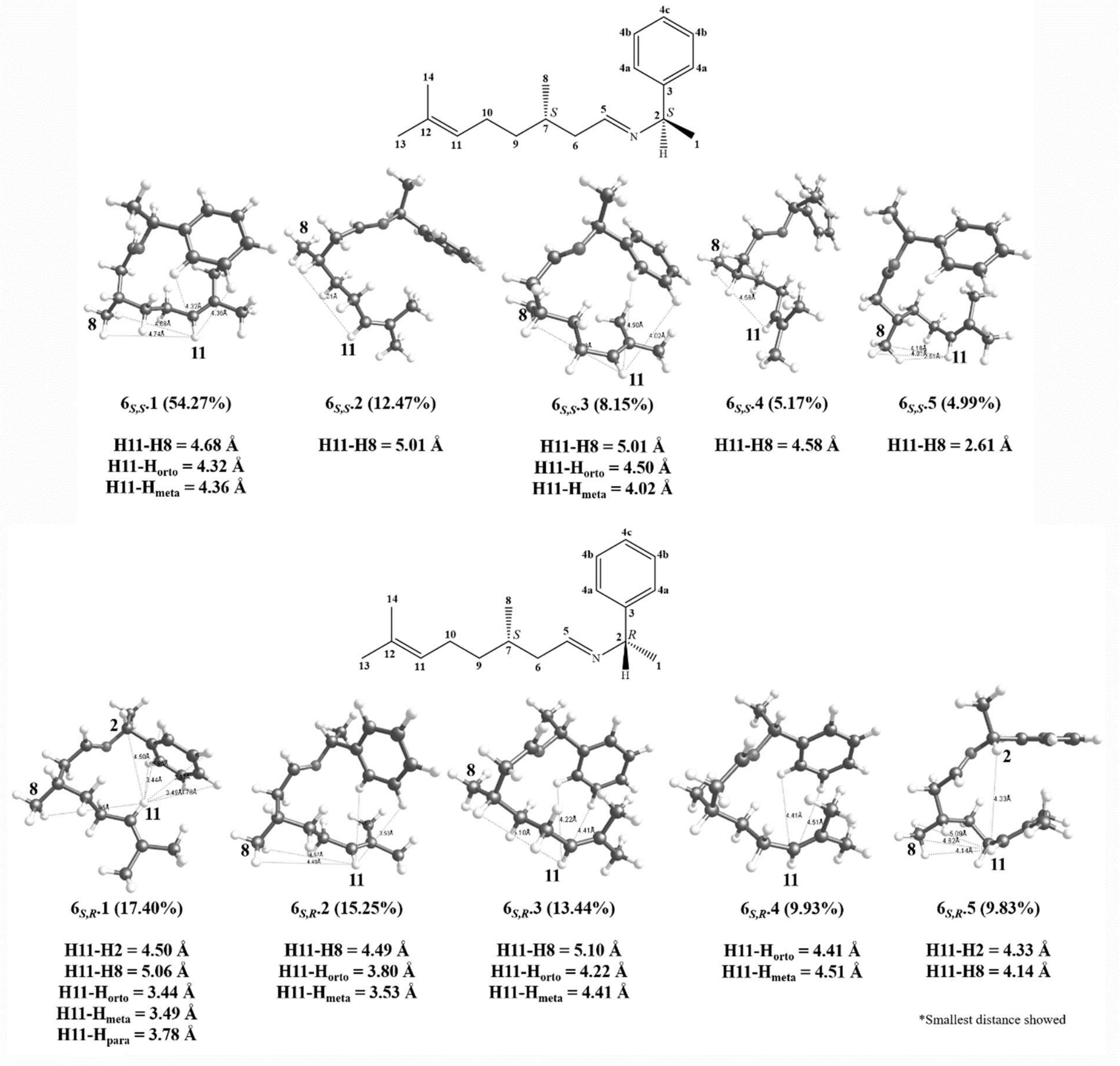

Figure S11. Five most stable conformers distribution of the diastereoisomer **6** (*S,S* and *S,R*) using B3LYP-D3(BJ)/def2-TZVP(-f) level. In parentheses is the percentage of Boltzmann's population. Just below each conformer, there is the distance, less than 5 Å, between the hydrogens.

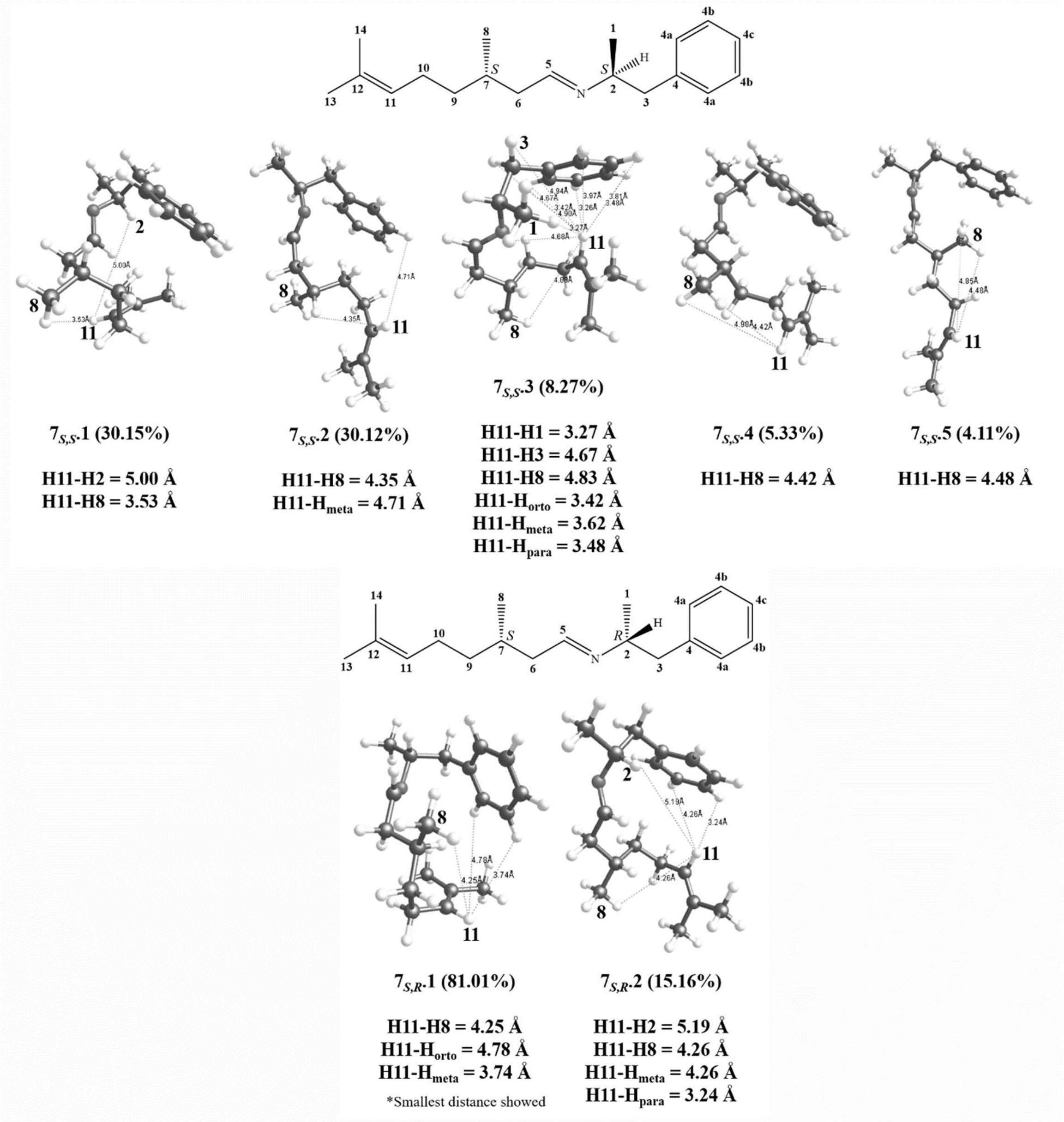

Figure S12. Five most stable conformers distribution of the diastereoisomer **7** (*S,S* and *S,R*) using B3LYP-D3(BJ)/def2-TZVP(-f) level. In parentheses is the percentage of Boltzmann's population. Just below each conformer, there is the distance, less than 5 Å, between the hydrogens.

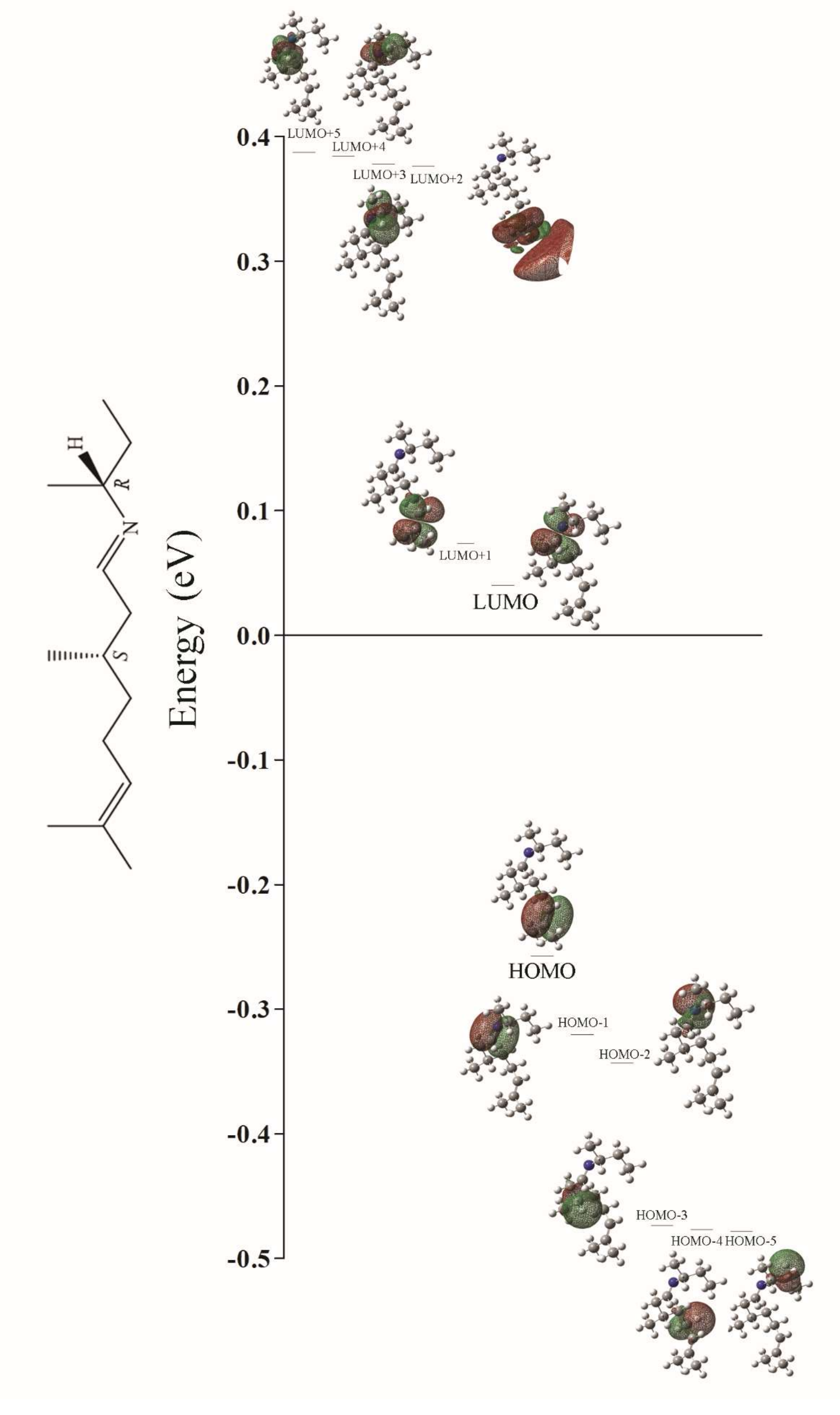

Figure S13. Energy diagram of the molecular orbitals of the diastereomeric imine $\mathbf{5}_{S,R}$.

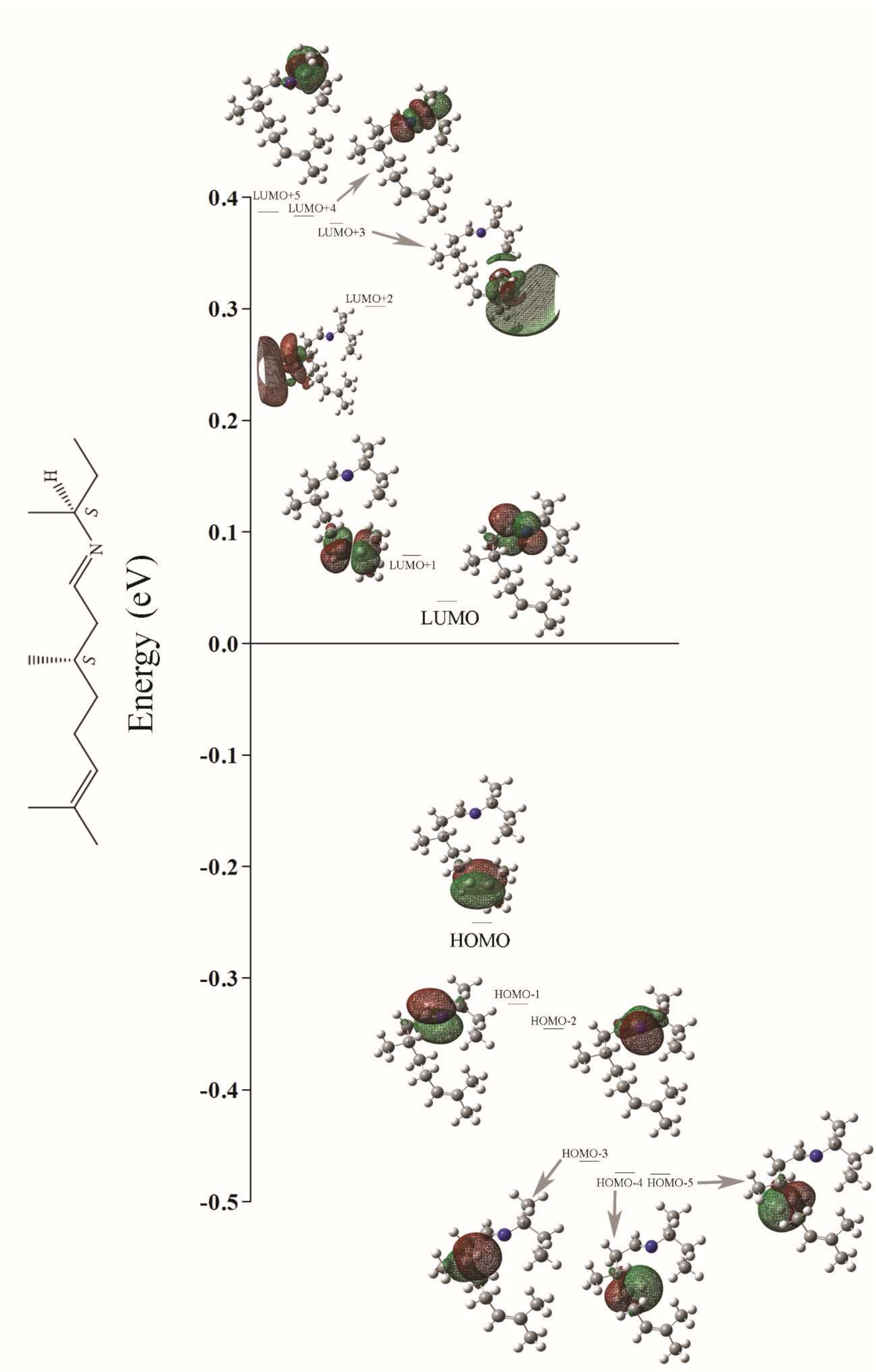

Figure S14. Energy diagram of the molecular orbitals of the diastereomeric imine $\mathbf{5}_{S,S.}$

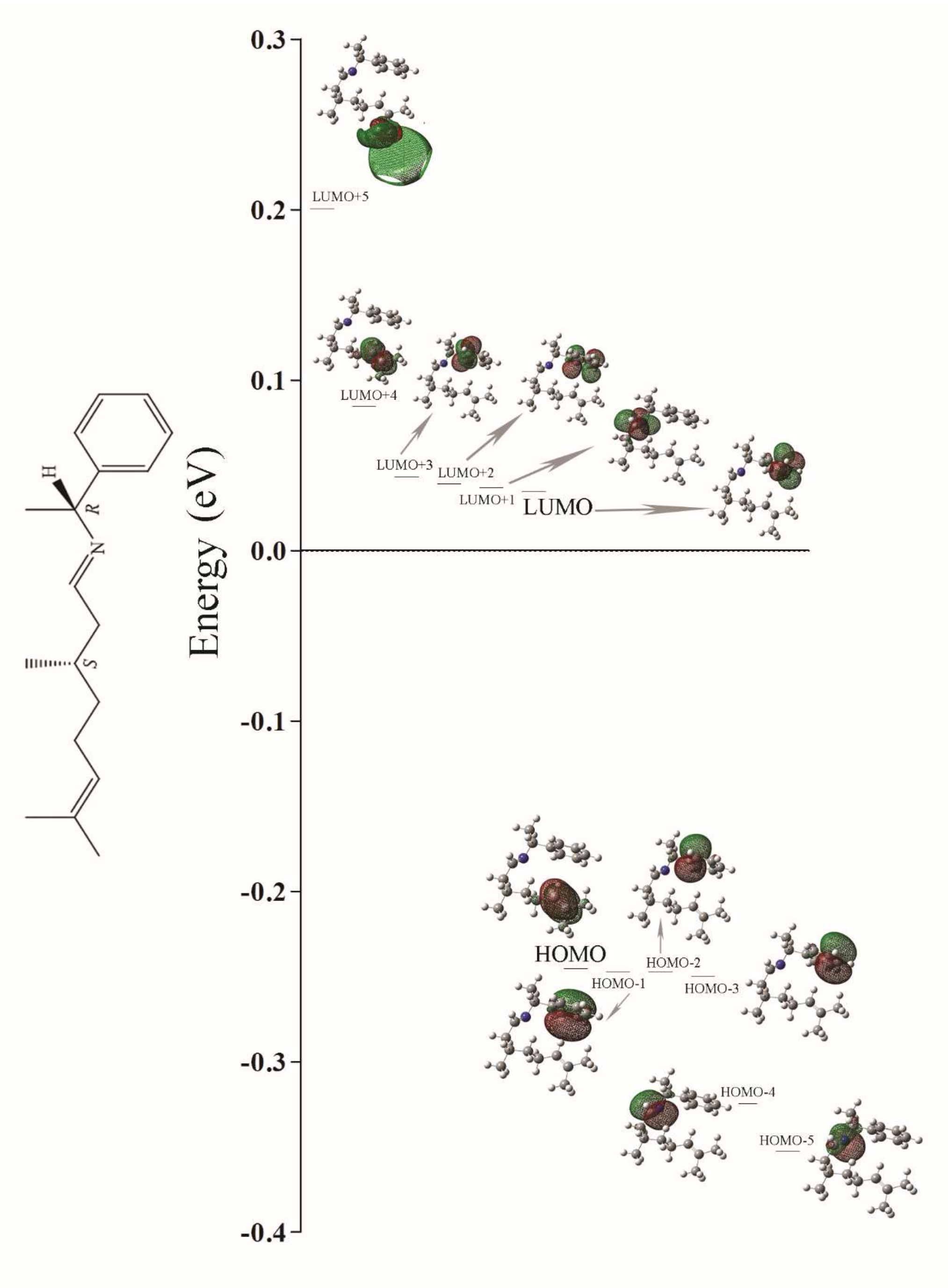

Figure S15. Energy diagram of the molecular orbitals of the diastereomeric imine $\mathbf{6}_{S,R}$.

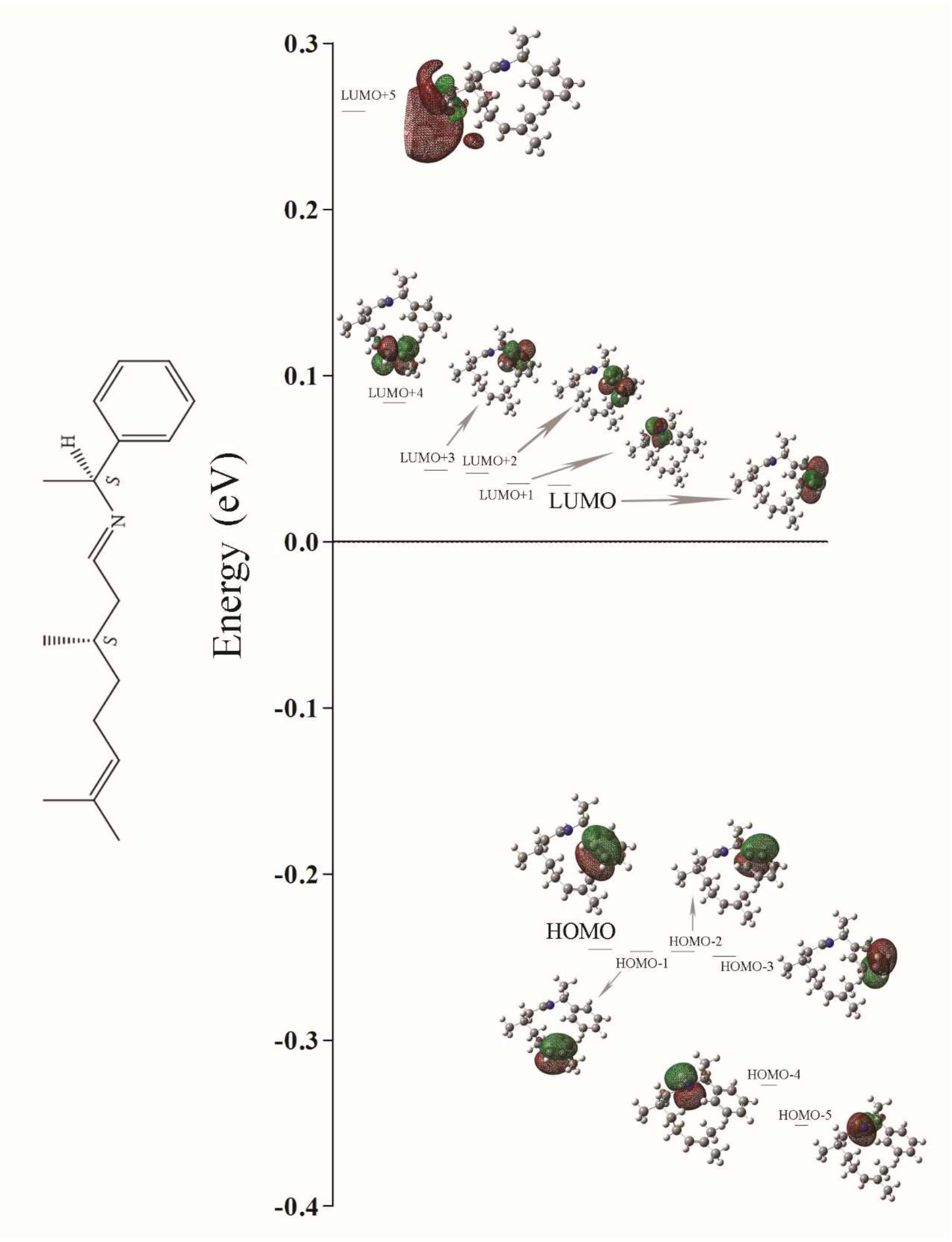

Figure S16. Energy diagram of the molecular orbitals of the diastereomeric imine $\mathbf{6}_{S,S.}$

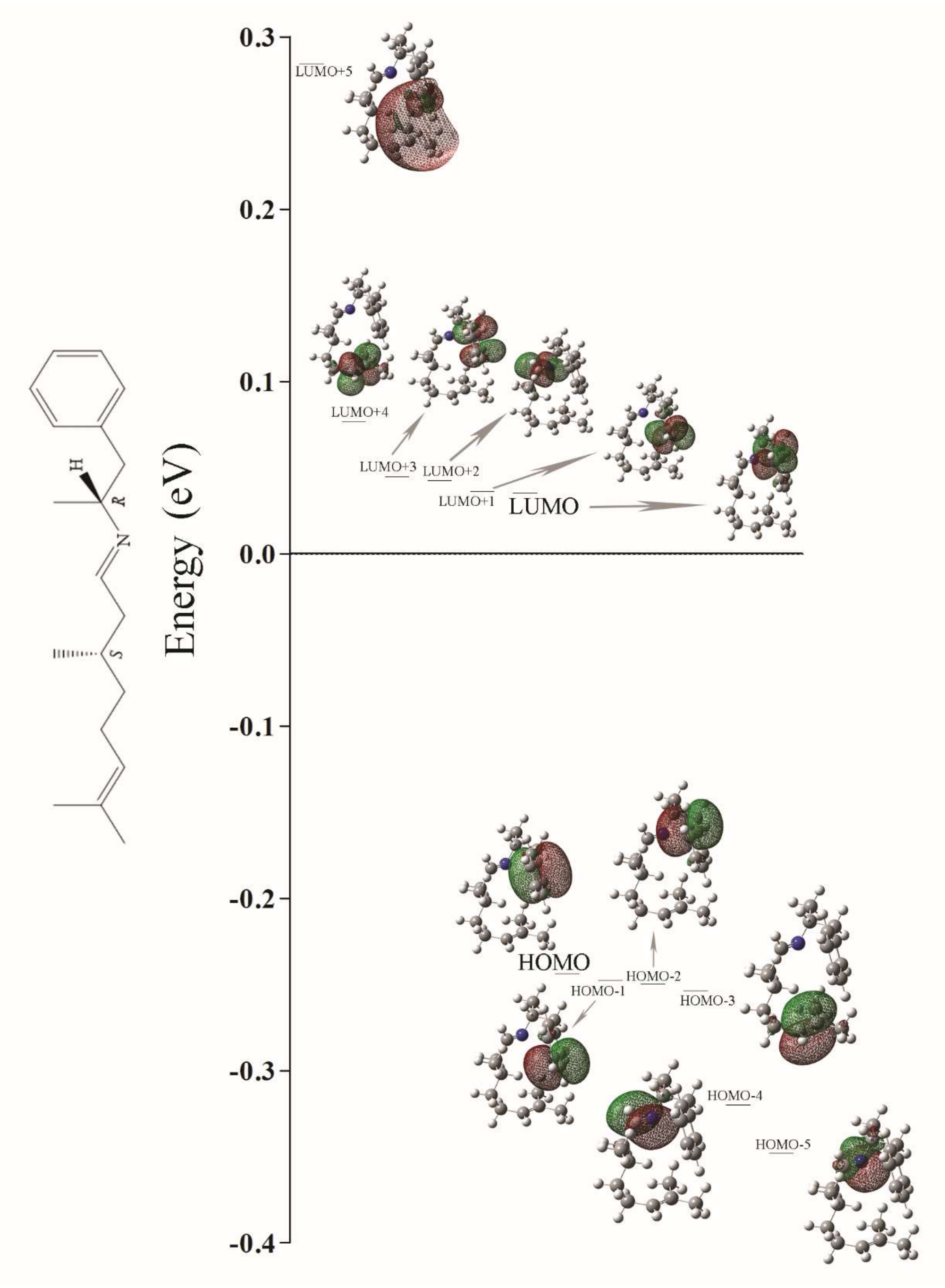

Figure S17. Energy diagram of the molecular orbitals of the diastereomeric imine $\mathbf{7}_{S,R}$.

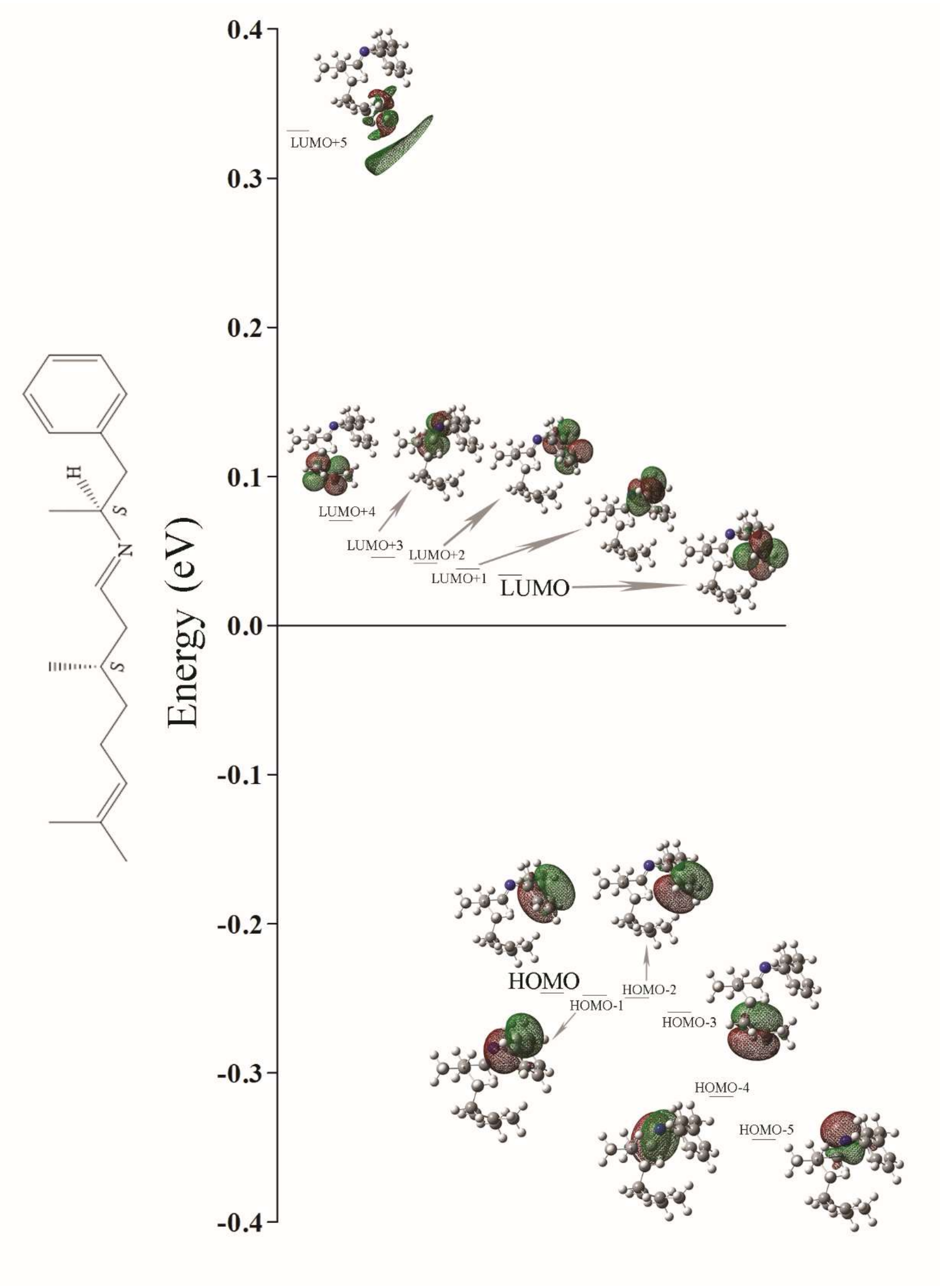

Figure S18. Energy diagram of the molecular orbitals of the diastereomeric imine $\mathbf{7}_{S,S.}$
